# Supplementary material for: In Vivo Reflectance Confocal Microscopy as a Response Monitoring Tool for Actinic Keratoses Undergoing Cryotherapy and Photodynamic Therapy
Source: Cancers (Basel). 2021 Oct 31;13(21):5488. doi: 10.3390/cancers13215488 (PMC8583298; doi:10.3390/cancers13215488)
Supplement: Supplementary file 1 [file cancers-13-05488-s001.zip › cancers-1401615-supplementary.pdf]

cancers

MDPI

Supplementary Materials: In Vivo Reflectance Confocal Microscopy as a Response Monitoring Tool for Actinic Keratoses Undergoing Cryotherapy and Photodynamic Therapy

Clara Curiel-Lewandrowski, Caitlyn N. Myrdal, Kathylynn Saboda, Chengcheng Hu, Edith Arzberger, Giovanni Pellacani, Franz Josef Legat, Martina Ulrich, Petra Hochfellner, Margaret C. Oliviero, Paola Pasquali, Melissa Gill and Rainer Hofmann-Wellenhof

Table S1. Percent prevalence among reads at baseline.

Skin Layer	RCM Parameter	PD skin	Grade 1 AK	Grade 2 AK	All AK
Stratum Corneum	Parakeratosis	27.7%	69.2% **	67.4% **	68.4% **
	Hyperkeratosis	12.8%	60.4% **	72.7% **	66.3% **
	Stratum Corneum Disruption	26.1%	71.2% **	75.5% **	73.3% **
Epidermis	Atypical Honeycomb Pattern	69.6%	98.2% **	98.1% **	98.2% **
	Round Nucleated Cells	9.1%	23.2%	14.0% *	18.9%
	Disarranged Epidermal Pattern	16.1%	50.9% **	60.8% **	55.6% **
	Presence of Inflammatory Epidermal Cells	38.9%	55.4% *	60.0%	57.5% *
	Presence of Dendritic Cells	13.0%	10.9%	14.3%	12.5%
Dermis	Inflammatory Infiltrate Dermis	32.7%	63.6%	47.1% *	55.7% *
	Solar Elastosis	78.4%	92.5%	84.8%	88.9%
	Round Blood Vessels	61.8%	69.6% *	75.5%	72.5% *
	Polymorphous Blood Vessels	16.4%	41.1% *	35.3% *	38.3% **

Significance compared to PD skin. *p < 0.05, **p < 0.01.

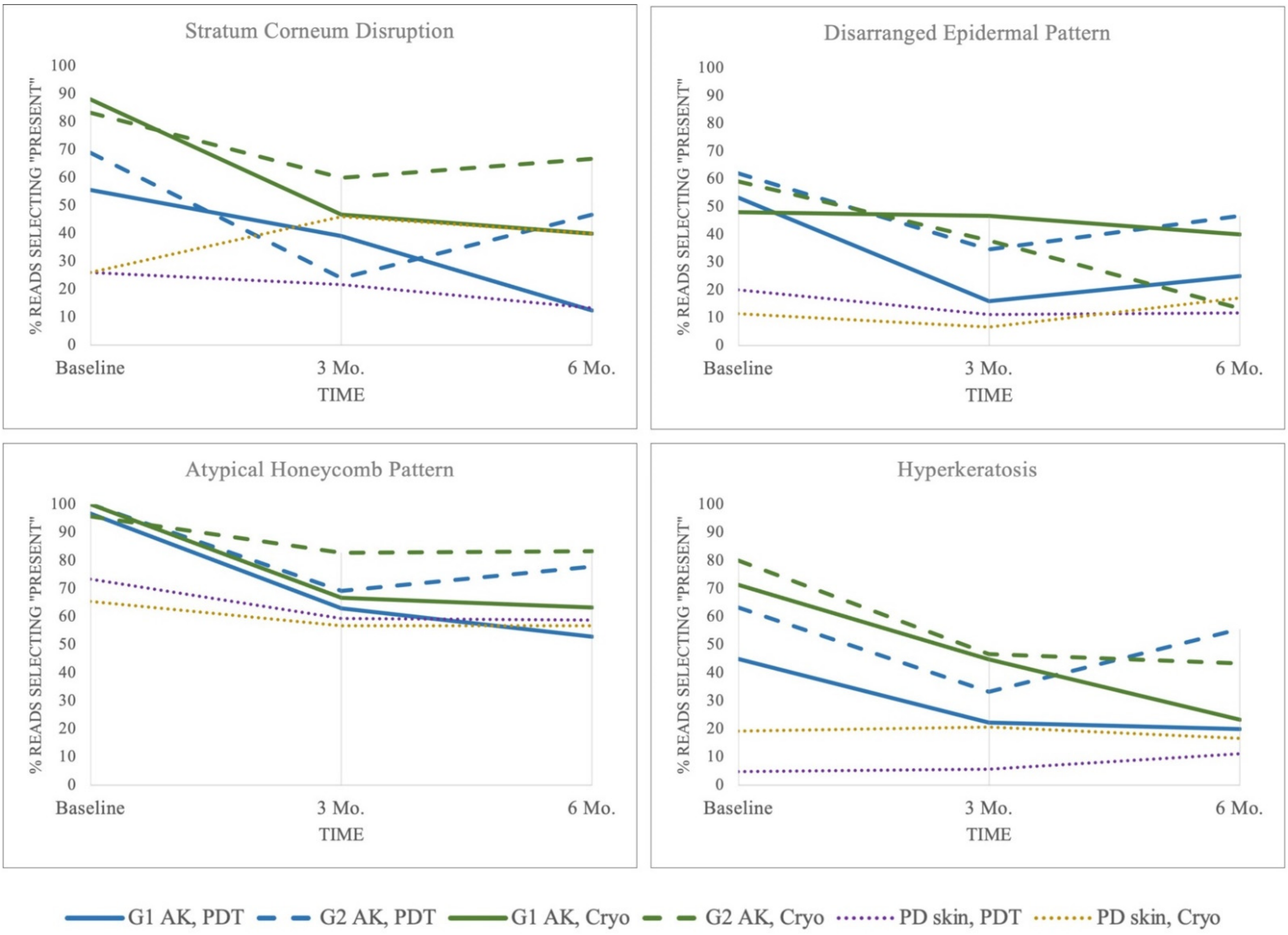

Figure S1. Graphical representation of key RCM criteria over time.
